# Influence of heart rate correction formulas on QTc interval stability

**DOI:** 10.1038/s41598-021-93774-9

**Published:** 2021-07-12

**Authors:** Irena Andršová, Katerina Hnatkova, Martina Šišáková, Ondřej Toman, Peter Smetana, Katharina M. Huster, Petra Barthel, Tomáš Novotný, Georg Schmidt, Marek Malik

**Affiliations:** 1 Department of Internal Medicine and Cardiology, Faculty of Medicine, University Hospital Brno, Masaryk University, Jihlavská 20, 625 00 Brno, Czech Republic; 2 National Heart and Lung Institute, Imperial College, ICTEM, Hammersmith Campus, 72 Du Cane Rd, Shepherd’s Bush, London, W12 0NN England, UK; 3 Wilhelminenspital der Stadt Wien, Montleartstraße 37, 1160 Vienna, Austria; 4 Klinikum rechts der Isar, Technische Universität München, Ismaninger Straße 22, 81675 Munich, Germany; 5 Department of Internal Medicine and Cardiology, Faculty of Medicine, Masaryk University, Jihlavská 20, 625 00 Brno, Czech Republic

**Keywords:** Biomarkers, Cardiology, Medical research

## Abstract

Monitoring of QTc interval is mandated in different clinical conditions. Nevertheless, intra-subject variability of QTc intervals reduces the clinical utility of QTc monitoring strategies. Since this variability is partly related to QT heart rate correction, 10 different heart rate corrections (Bazett, Fridericia, Dmitrienko, Framingham, Schlamowitz, Hodges, Ashman, Rautaharju, Sarma, and Rabkin) were applied to 452,440 ECG measurements made in 539 healthy volunteers (259 females, mean age 33.3 ± 8.4 years). For each correction formula, the short term (5-min time-points) and long-term (day-time hours) variability of rate corrected QT values (QTc) was investigated together with the comparisons of the QTc values with individually corrected QTcI values obtained by subject-specific modelling of the QT/RR relationship and hysteresis. The results showed that (a) both in terms of short-term and long-term QTc variability, Bazett correction led to QTc values that were more variable than the results of other corrections (*p* < 0.00001 for all), (b) the QTc variability by Fridericia and Framingham corrections were not systematically different from each other but were lower than the results of other corrections (*p*-value between 0.033 and < 0.00001), and (c) on average, Bazett QTc values departed from QTcI intervals more than the QTc values of other corrections. The study concludes that (a) previous suggestions that Bazett correction should no longer be used in clinical practice are fully justified, (b) replacing Bazett correction with Fridericia and/or Framingham corrections would improve clinical QTc monitoring, (c) heart rate stability is needed for valid QTc assessment, and (d) development of further QTc corrections for day-to-day use is not warranted.

## Introduction

Good clinical practice requires the assessment of heart rate corrected QTc interval in a variety of situations^1^. As well known, QTc assessment and/or monitoring is mandated when administering drugs recognised to affect myocardial repolarisation and to potentially induce torsade de pointes tachycardia^2^; when diagnosing the sources of syncopal and/or pre-syncopal episodes^3^; when considering the proarrhythmic consequences of treatment-induced electrolyte abnormalities^4,5^; when screening relatives of patients with recognised repolarisation channelopathy^6^ or of unexplained sudden death victims^7^; and in a further spectrum of other circumstances and conditions. To support this practice, some healthcare providers stipulate corresponding guidelines. QTc prolongation assessment has also been the topic of proposed scoring systems^8^.

Reports have also been published evaluating the effectiveness of the suggested good clinical practice^9–11^. Regrettably, it has been reported that while the introduction of guidelines and monitoring schemes increases the awareness of the proarrhythmic potentials, therapeutic implications of prolonged QTc interval, such as therapy changes and/or more intensive monitoring, are not necessarily systematically implemented^12,13^.

Speculations of the reasons for these discrepancies between healthcare guidelines and day-to-day practice include, among others, the intra-subject variability of QTc intervals that does not allow monitoring QTc changes and/or detecting QTc abnormalities with sufficiently robust accuracy^14^. It is surely true that QTc interval changes may reflect not only treatment-related and channelopathy-based repolarisation abnormalities^2^ but also electrolyte differences^15^, fever^16^, hormonal changes^17^, and alteration in autonomic and central nervous status^18^. Nevertheless, it is also apparent that imprecisions of QT interval measurement and inaccuracies of its heart rate correction may substantially contribute to an increased variability of measured QTc values^19^.

Electrocardiographic experience from clinical pharmacology studies suggests that intra-subject QTc variability is substantially reduced not only by systematic QT interval measurements but also, and largely, by using subject-specific heart rate corrections^20^. These observations are of little help in clinical practice when baseline subject-specific QT/RR profiles are not and cannot be available. Nevertheless, scores of different heart rate corrections have previously been proposed^21,22^. Although it has repeatedly been shown that some of these corrections are superior to others at eliminating the heart rate dependency of QTc intervals^22^, the differences between corrections in terms of intra-subject QTc variability have not been systematically investigated.

With this in mind, we conducted a study that utilised data in healthy volunteers and investigated whether and how different heart rate corrections influence the intra-subject QTc variability and whether and how this variability might be reduced by recordings strategies and conditions.

## Methods

### Investigated population and long-term electrocardiographic recordings

Clinical pharmacology studies conducted at 4 different locations enrolled 539 healthy volunteers (259 females) aged, on average, 33.3 ± 8.4 years (no statistical age differences between females and males). Before study enrolment, all the volunteers had a normal standard electrocardiogram (ECG) and all had normal clinical investigation. Standard inclusion and exclusion criteria mandated for Phase I pharmacology studies^23^ were used including negative tests for recreational substances and negative pregnancy tests for females. All the source studies were ethically approved by the institutional ethics bodies (Parexel in Baltimore; California Clinical Trials in Glendale; PPD in Austin; and Spaulding in Milwaukee). Consistent with Helsinki declaration, all subjects gave informed written consent to study participation and to research use of data collected during the studies. As only drug-free data were analysed in the present investigation and as there were no differences in clinical handling of study subjects at the investigation sites during the drug-free days, further details of the source clinical studies are of no relevance.

Demographic descriptors were available for all study subjects. Body mass index (BMI) was defined as body weight in kilograms divided by the squared body height in metres; lean body mass (LBM) was calculated as LBM = 0.29569 × (body weight[kg]) + 41.813 × (body height[m]) − 43.2933 for females, and LBM = 0.3281 × (body weight[kg]) + 33.929 × (body height[m]) − 29.5336 for males^24^.

In all volunteers, repeated long-term 12-lead Holter ECG recordings were obtained covering the full day-time periods. These recordings included ECG collected during days when the subjects were on no medication. The subjects were also not allowed to smoke and/or consume alcohol or caffeinated drinks. The principal analysis of the investigation reported here concerned only the first day of the source clinical pharmacology studies which made the data fully consistent across the clinical centres. The protocols of the source studies were also fully consistent with each other as far as handling of the volunteers during the drug-free baseline days was concerned. Among others, all the volunteers were fasting during the morning hours of the baseline days and their first meal of the day consisted of a standardised lunch, as mandated during the Phase I clinical studies. No high fat diet loading was used. During the day-time hours of the baseline days, the study subjects were also not permitted to sleep.

Using previously described technology^25,26^, multiple 10-s ECG segments were extracted from the long-term ECGs (see also further details in this text) and in each of these segments, QT interval was measured including repeated visual controls of all the measurements and assurance that corresponding ECG morphologies were interpreted in a consistent way^27^. The interval measurements were performed using representative median waveforms of the 10-s segments (sampled at 1000 Hz) with the superimposition of all 12 leads on the same isoelectric axis^28,29^. Exactly the same measurement organisation was applied to all source ECG data. This included, among others, the same algorithms for 10-s ECG segment extraction and for creation of the representative waveforms, the same system for initial QT measurement and consistency checks, and identical organisation of visual control and manual correction of the QT measurements.

Also using previously described techniques and procedures, individual curvatures of QT/RR patterns (i.e. how much the QT interval changes in response to underlying heart rate changes) including the individual profiles of QT/RR hysteresis (i.e. how quickly the QT interval changes following a heart rate change) were constructed for each study subject^30,31^. In more detail, multiple measurements of QT intervals with the preceding history of RR intervals were available in each subject. This allowed, in each subject separately, to construct a curvilinear regression ${\text{QT}}_{{\text{i}}} = {\upchi } + {\upphi }\left( {1 - \left( {\widetilde{{{\text{RR}}}}_{{\text{i}}} } \right)^{{\upgamma }} } \right) + {\upvarepsilon }_{{\text{i}}}$ where ${\text{QT}}_{{\text{i}}} ~$ are individual QT interval measurements in seconds, $\widetilde{{{\text{RR}}}}_{{\text{i}}}$ are RR interval durations (in seconds) representing the hysteresis corrected^31^ heart rate underlying the ${\text{QT}}_{{\text{i}}} ~$ measurement, ${\upchi }$ and ${\upphi }$ are intercept and slope of linear regression, ${\upvarepsilon }_{{\text{i}}}$ are normally distributed zero centred errors, and the parameter ${\upgamma }$ is individually optimised so that it leads to the lowest regression residual. These were converted into individual heart rate correction of the measured QT intervals, QTcI = QT $+ {\text{ }}{\upphi }\left( {1 - \widetilde{{RR}}^{{\upgamma }} } \right)$. Individually corrected QTcI values for each of the measured ECG segments were obtained in this way. In further investigation, the QTcI values were used as the “gold standard” control data for the comparison of different heart rate correction formulas.

### Heart rate correction formulas

To investigate the influence of different generic heart rate corrections on the character and variability of QTc data, 10 previously proposed correction formulas were considered. In order to model standard clinical situations, the average of all RR intervals found in the measured 10-s ECG sample (or the corresponding heart rate) was used in all the corrections. The following corrections were used:

- Bazett (BAZ)^32^ QTc = QT/RR^1/2^
- Fridericia (FRI)^33^ QTc = QT/RR^1/3^
- Dmitrienko (DMI)^34^ QTc = QT/RR^0.413^
- Framingham (FRA)^35^ QTc = QT + 0.154 × (1 − RR)
- Schlamowitz (SCH)^36^ QTc = QT + 0.205 × (1 − RR)
- Hodges (HOD)^37^ QTc = QT + 0.00175 × (HR − 60)
- Ashman (ASH)^38^ QTc = QT/log_10_(10 × (RR + 0.07)) × log_10_(10.7)
- Rautaharju (RAU)^39^ QTc = QT + 0.24251 − 0.434 × e^–0.0097 × HR^
- Sarma (SAR)^40^ QTc = QT − 0.04462 + 0.664 × e^–2.7 × RR^
- Rabkin (RAB)^41^ QTc = [$\widehat{{QT}}$(60,0,50.3) + 1000 × QT − $\widehat{{QT}}$(HR,*sex*,*age*)]/1000

In these formulas, QT and averaged RR intervals are measured in seconds, HR is the heart rate in beats per minute (bpm) corresponding to the measured averaged RR interval, *sex* is the numerical sex indicator of the sex of the subject, and *age* is the age of the subject in years. In the formula by Rabkin at al^41^, $\widehat{{QT}}$ is a published spline function^42^ dependent on the HR value with additional sex and age components. Specifically, the formula by Rabkin et al. denotes $\widehat{{QT}}$(HR,sex,age) = $\vartheta _{0} - \mathop \sum \limits_{{i = 1}}^{7} \vartheta _{i} {\mathcal{B}}_{i} \left( {{\text{HR}}} \right) +$ 9.35 × *sex* + 0.18 × *age* (in milliseconds), where ${\mathcal{B}}_{i}$ are composed of polynomial fractions that depend only on heart rate, *ϑ*_*i*_ are numerical coefficients, *age* is the age of the subject in years, and *sex* is an indicator 1 and 0 for females and males, respectively^42^. Contrary to other formulas that aim at correcting the QT intervals to the heart rate of 60 bpm (and thus, at the heart rate of 60 bpm, lead to QTc = QT) the formula by Rabkin et al. normalizes the measured QT intervals to a distribution in a 50.3-year male at 60 bpm (derived from the data analysed by Rabkin et al.^41,42^). Intentionally, this formula aims at eliminating the difference between females and males by reporting the QTc values in females reduced by 9.35 ms and also aims at eliminating QTc prolongation with advanced age.

The measured QT and RR intervals as well as the QTc intervals provided by the formulas that we listed are expressed in seconds. Note that some of the original publications showed the formulas for QT and QTc in milliseconds rather than in seconds, this is also the reason why we used the 1000 coefficient in the Rabkin et al. formula implementation since the spline function $\widehat{{QT}}$ provides QTc estimates in milliseconds—in the implementation of other formulas, the coefficients shown above have been adopted for the millisecond-second conversion where necessary.

We selected these formulas from the large spectrum of previously made proposals to investigate different mathematical forms as well as to include both older and more recent suggestions.

In the comparisons of the correction possibilities, we applied all these formulas as well as the individual-specific QTcI formula to the same set of the ECGs. That is, for each measured 10-s ECG segment, we obtained altogether 11 QTc values. Note also that while the generic formulas (BAZ through RAB) used averages of RR intervals in 10-s ECG recordings, the QTcI formula used hysteresis corrected RR intervals. We used this approach to increase the accuracy of QTcI values.

### Electrocardiographic data

#### Time-points

The protocol of all source studies included 27 time-points during which the subjects were kept motionless in undisturbed stable positions without any external interference. Each of the time-points lasted for 10 min and during the last 5 min, QT interval measurements were made in 5 different 10-s ECG segments that were separated by at least 20 s of each other. As described subsequently, QTc variability and accuracy was investigated among these QT (and corresponding RR) interval measurements.

#### Exclusion of post-prandial changes

Since meal intake is known to influence QT (and QTc) interval duration^43,44^, separate QTc analysis was performed using only segments of study time-points that occurred during the morning hours while the subjects were fasting.

#### Stable time-points

**Figure 1:**
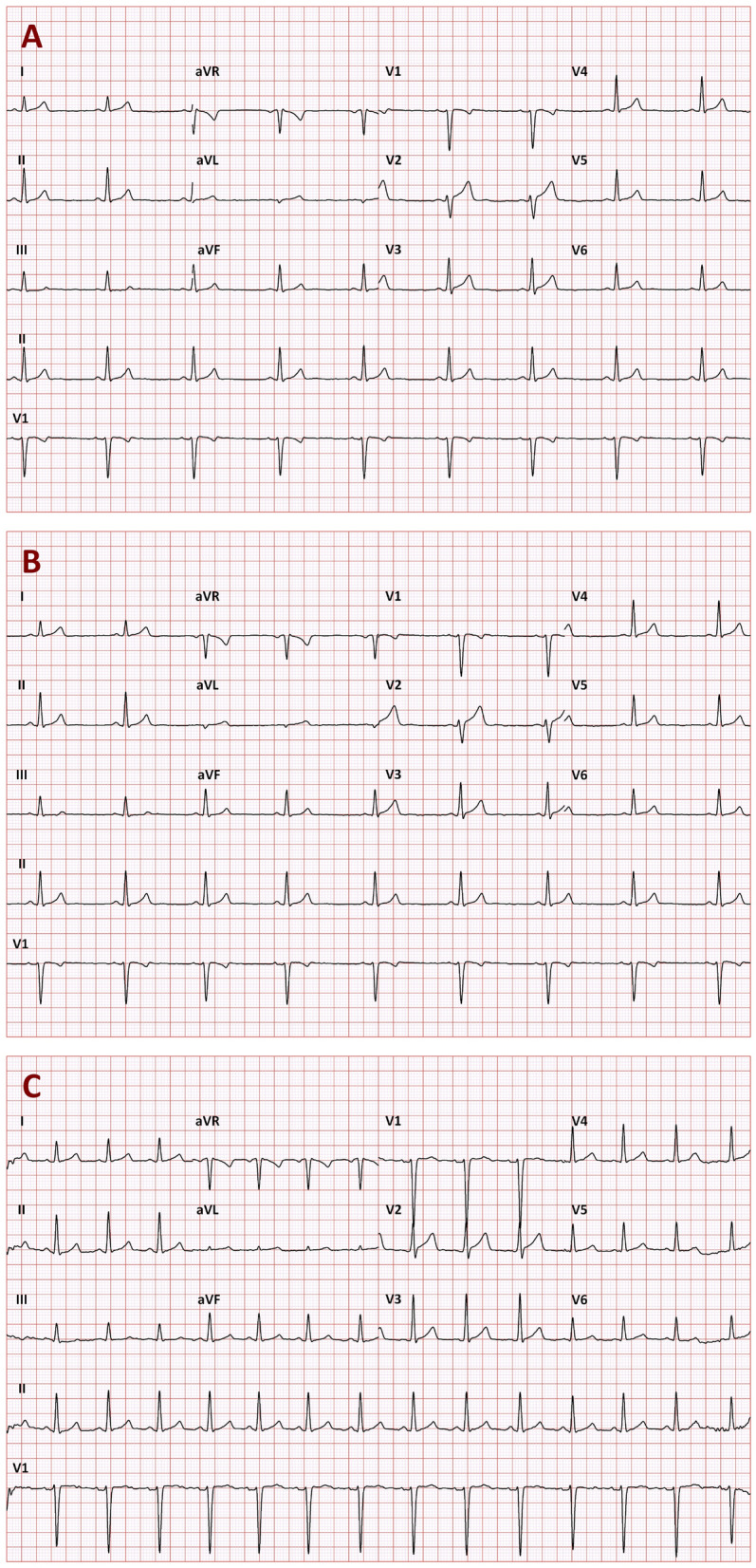
Example of three 10-s ECGs recorded in a 30-year old male in a short succession while the subject was kept in a strict motionless position. On the baseline drug-free day, the recordings A, B, and C were recorded at 13:34:38, 13:34:58, and 13:35:28, respectively. Their 10-s heart rates were 52.7, 52.6, and 85.9 bpm, and their uncorrected QT intervals were 405, 405, and 395 ms, respectively. When individual QT/RR hysteresis profile was incorporated into the assessment, the heart rates underlying the QT interval duration were 53.6, 53.4, 58.3 bpm which led to individually corrected QTcI intervals of 391.0, 390.7, and 391.5 ms, respectively. However, when the 10-s heart rates were used to correct the QT intervals, Bazett correction led to QTc values of 379.4, 379.2, and 472.7 ms, respectively (92 ms QTc increase). Fridericia correction let to QTc values of 387.7, 387.6, 445.2 ms, respectively (58 ms QTc increase). These QTc increases were erroneous since they resulted from the disassociation of the QT interval duration from the underlying heart rate. Similar erroneous QTc increases were found with all the investigated corrections formulas (QTc increase of 74, 58, 81, 48, 74, 62, 60, and 61 ms for the Dmitrienko, Framingham, Schlamowitz, Hodges, Ashman, Rautaharju, Sarma, and Rabkin corrections, respectively).

The QT/RR hysteresis is a known problem affecting the QTc assessment if the simultaneous RR interval measurement does not correspond to the underlying heart rate that influences the QT interval duration^31,45^. While keeping the study subjects in undisturbed stable positions eliminates all physical activity that might cause heart rate changes, other heart rate variations (e.g. due to mental processes) cannot be removed^46^. Heart rate variations might therefore occur even during the stable undisturbed positions. During such episodes, substantial QT/RR hysteresis problems might occur (example in Fig. 1).

Separate analyses of QTc variability and accuracy were therefore conducted considering only data from time-point windows during which the heart rate span (defined as the difference between the fastest and slowest heart rate of the measured 10-s ECG segments) was below 5 bpm. The same restriction was also additionally applied to fasting time-points.

#### Additional ECG measurements

In addition to the 10-s ECG segments extracted from the per-protocol study time-points, further extractions of additional 10-s segments (all preceded by stable heart rates) were made with the aim of covering different stable heart rates encountered during each recording. As described further, these additional QT and RR measurements were combined with the extractions from all time-points and used in the analysis of whether the heart rate correction formulas eliminated the influence of heart rate on the QTc intervals.

### Data investigations

#### Population characteristics

Since the variability and accuracy of QTc values by different formulas is likely influenced by the ECG-related characteristics of the investigated population, we have investigated these properties independently of any of the generic correction formulas.

In each subject, the mean QTcI interval and the standard deviation (SD) of the QTcI intervals measured in all the measured 10-s ECG segments as described in the previous section, and the slope of the log-linear QT/RR relationship (for hysteresis corrected RR intervals) were related to age, BMI, and LBM. (Note that the log-linear model of the QT/RR relationship is not an individually optimal expression of the QT/RR relationship^47^ but we used it since it corresponds to the mathematics of the first 3 of the evaluated formulas.)

#### Short-term QTc variability

Short-term variability of the QTc values reported by the different formulas was investigated in the windows of individual time-points of the original studies. That is, each separate time-point defined a 5-min window during which five repeated QT and 10-s heart rate measurements were made. From these data, SD of the QTc values were obtained and related to the range of the measured heart rates, i.e. to the difference between the fastest and the slowest of the five 10-s heart rate measurements. The intra-subject dependencies of the SD of the QTc values on the heart rate ranges were compared between different correction formulas.

#### Day-time QTc variability

In each subject, SD of all QTc values were compared between the correction formulas when considering ECG measurements in (a) all ECG extractions from the Holter recordings (i.e. including those that were made outside the per-protocol study time-points), (b) all ECG extractions from per-protocol study time-points, (c) ECG extractions from per-protocol study time-points while the subjects were fasting during the morning of the baseline day, (d) all ECG extractions from study time-points that showed heart rate range below 5 bpm, and (e) ECG extractions from fasting study time-points that showed heart rate range below 5 bpm.

#### Difference between general and individual QTc corrections

By definitions, the individual corrections create QTcI values that are, over the entire recording of the given subject, independent of the underlying heart rates. To investigate how much the generic correction formulas differed from this ideal situation, the slopes of linear regressions between the QTc values by the different corrections and the corresponding 10-s RR intervals were calculated in each subject. For this purpose, all ECG extractions from the Holter recordings were used. While in some previous studies, the slopes and correlations between QTc values and the RR intervals of the underlying heart rates were investigated over complete population of different subjects or patients^48^, we have evaluated the linear QTc/RR regressions in each subject separately. This was because the optimisation of a correction formula over a population of different subject may (and frequently does) lead to corrections that are systematically biased in all or a majority of subjects of the population if applied to each of them separately^49,50^.

The differences between QTc values by the different corrections and the corresponding QTcI intervals were evaluated, in each subject, considering the (a) ECG extractions from all per-protocol study time-points, (b) ECG extractions from fasting per-protocol study time-points, (c) ECG extractions from study time-points with heart rate range below 5 bpm, and (d) ECG extractions from fasting study time-points with heart rate range below 5 bpm.

### Statistics and data presentation

Descriptive data are presented as means ± SD. The QTc variability and QTc—QTcI differences are presented as population medians, inter-quartile ranges, and 90 percent ranges, that is the intervals between the 5th and 95th population percentile. Where appropriate, linear regressions were calculated together with the 95% bands of the regression lines. Comparisons between female and male subjects were based on two-sample two-tail t-tests assuming different variances of the compared samples. Within subject comparisons were based on paired two-sample t-tests. *P* value below 0.05 was considered statistically significant. Because of the inter-dependence of the data, no adjustment for multiplicity of testing was performed.

## Results

The data of the 539 healthy volunteers involved altogether 452,440 measurements of individual 10-s ECG samples.

### Population QTcI characteristics

**Figure 2:**
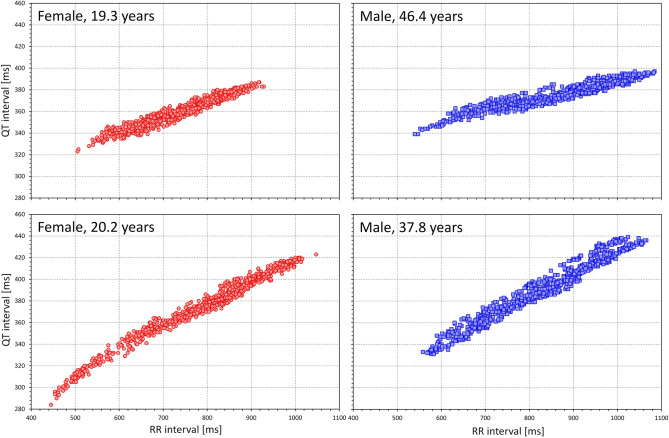
Examples of repeated intra-subject drug-free QT measurements related to the underlying (hysteresis corrected) RR intervals. The examples in females and males are shown with red and blue symbols, respectively, ages of the subjects are shown in the individual panels. Note the substantial differences of the slopes of the patterns.

As expected^51^, the individual QT/RR patterns differed substantially between study subjects (examples in Fig. 2). To characterise them we have considered intra-subject QTcI averages over (a) all time-points, (b) stable time-points, and (c) fasting time-points.

Also as expected, intra-subject averages of QTcI durations were longer in females compared to males^52^. When considering the data of all study time-points, the averaged QTcI values in females and males were 419.6 ± 13.3 and 399.6 ± 12.5 ms, respectively (*p* < 0.00001 for the comparison between sexes). The averaged intra-subject QTcI values of stable time-points (averages in females and males of 419.6 ± 13.3 and 399.6 ± 12.6, respectively) were not statistically different from the intra-subject averages of all time-points. Nevertheless, considering only fasting time-points, the intra-subject QTcI averages (averages in females and males of 421.5 ± 13.5 and 400.9 ± 12.7 ms, respectively) were marginally but statistically significantly prolonged compared to the intra-subject averages of all time-points (*p* < 0.00001 for pair-wise comparisons in both sexes). This was consistent with previous reports of post-prandial QTc shortening^43,44^. The intra-subject SD of QTcI values taken from all the time-points was also larger in females compared to males (4.75 ± 1.36 vs. 4.27 ± 1.16, *p* < 0.00001). In both sexes, these SD values were reduced by eliminating the post-prandial effects, and the statistical sex difference was reduced although it remained statistically significant (3.30 ± 1.40 vs. 3.05 ± 1.35 ms, *p* = 0.035). The full profile QT/RR patterns were also steeper in females compared to males^30,52,53^, with log-linear slopes of 0.366 ± 0.047 vs. 0.331 ± 0.045, *p* < 0.00001.

**Figure 3:**
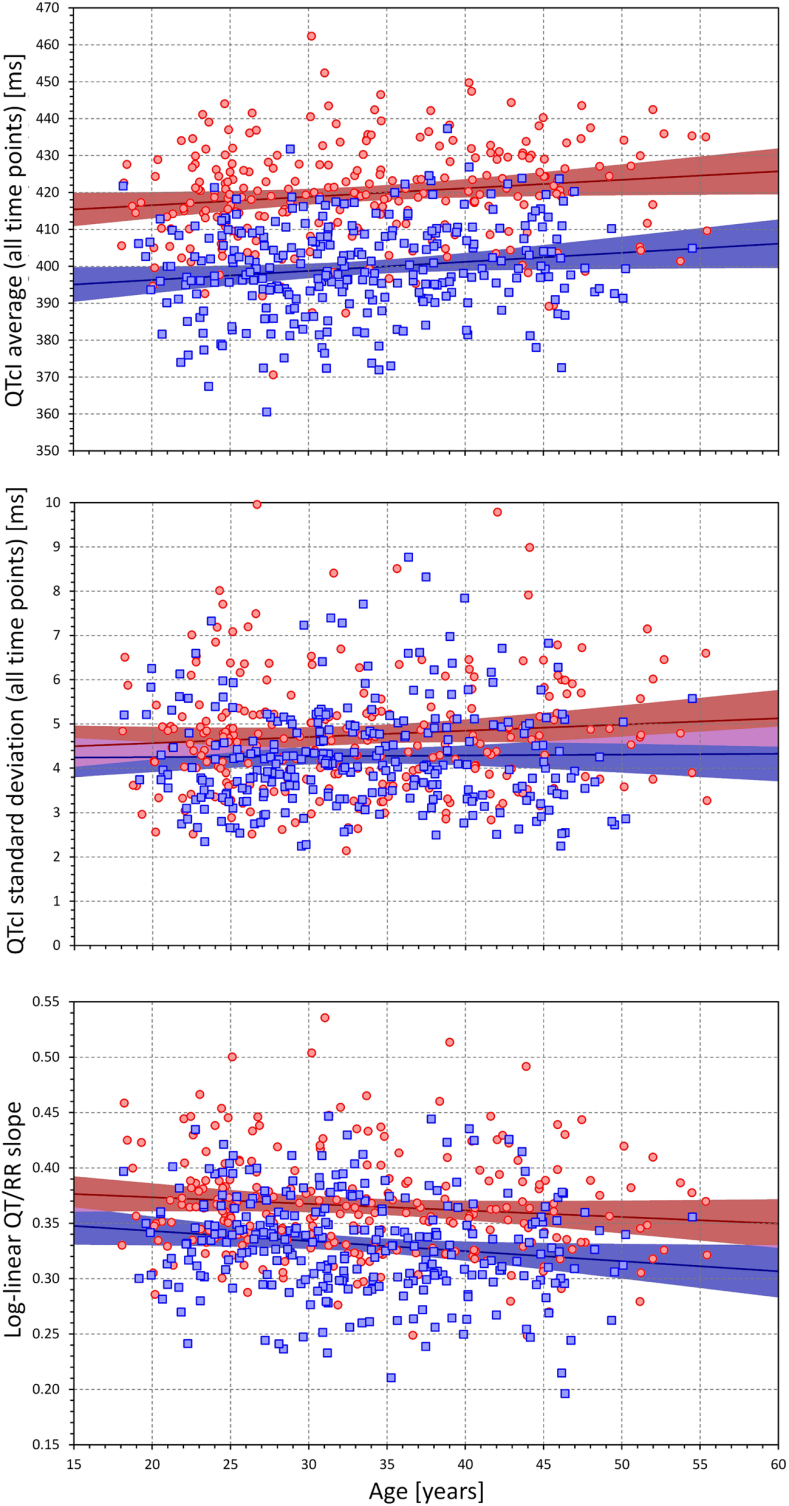
Scatter diagrams between age and mean QTcI values of all ECG measurements in all study time-points (top panel), standard deviation of QTcI values of all ECG measurements in all study time-points (middle panel), and subject-specific log-linear QT/RR slope (bottom panel). In each panel, the red circles and blue squares correspond to female and male subjects, respectively. The solid red and solid blue lines show the linear regressions between the age and the measured QT characteristics in females and males, respectively. The red shaded and blue shaded areas are the 95% confidence intervals of the regression lines; the violet areas are the overlaps between the confidence intervals of the sex-specific regressions. *ms *milliseconds.

**Figure 4:**
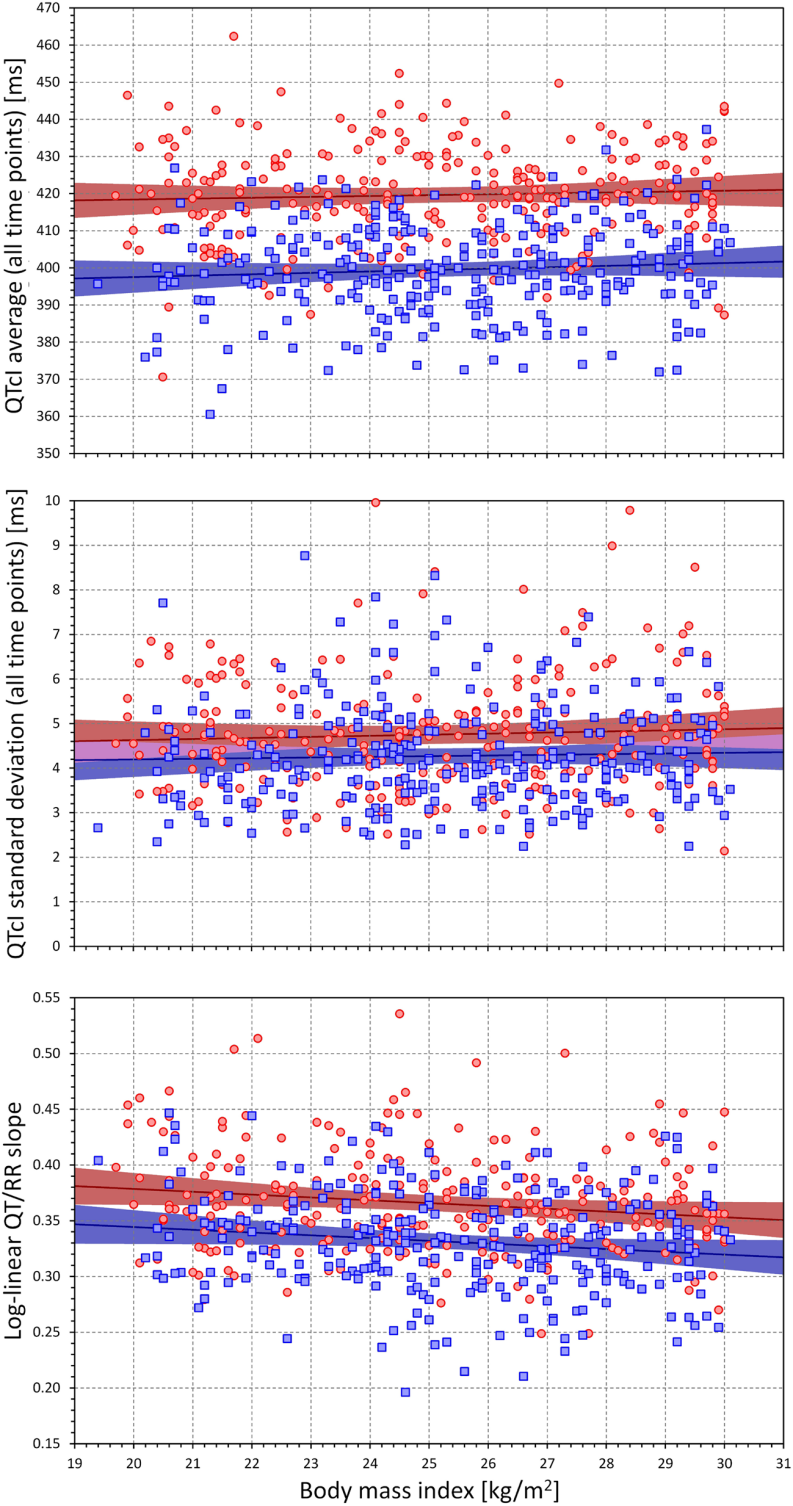
Scatter diagrams between the body mass index and mean QTcI values of all ECG measurements in all study time-points (top panel), standard deviation of QTcI values of all ECG measurements in all study time-points (middle panel), and subject-specific log-linear QT/RR slope (bottom panel). The meaning of symbols and Figure layout is the same as in Fig. 3. *kg* kilograms, *m* metre, *ms* milliseconds.

**Figure 5:**
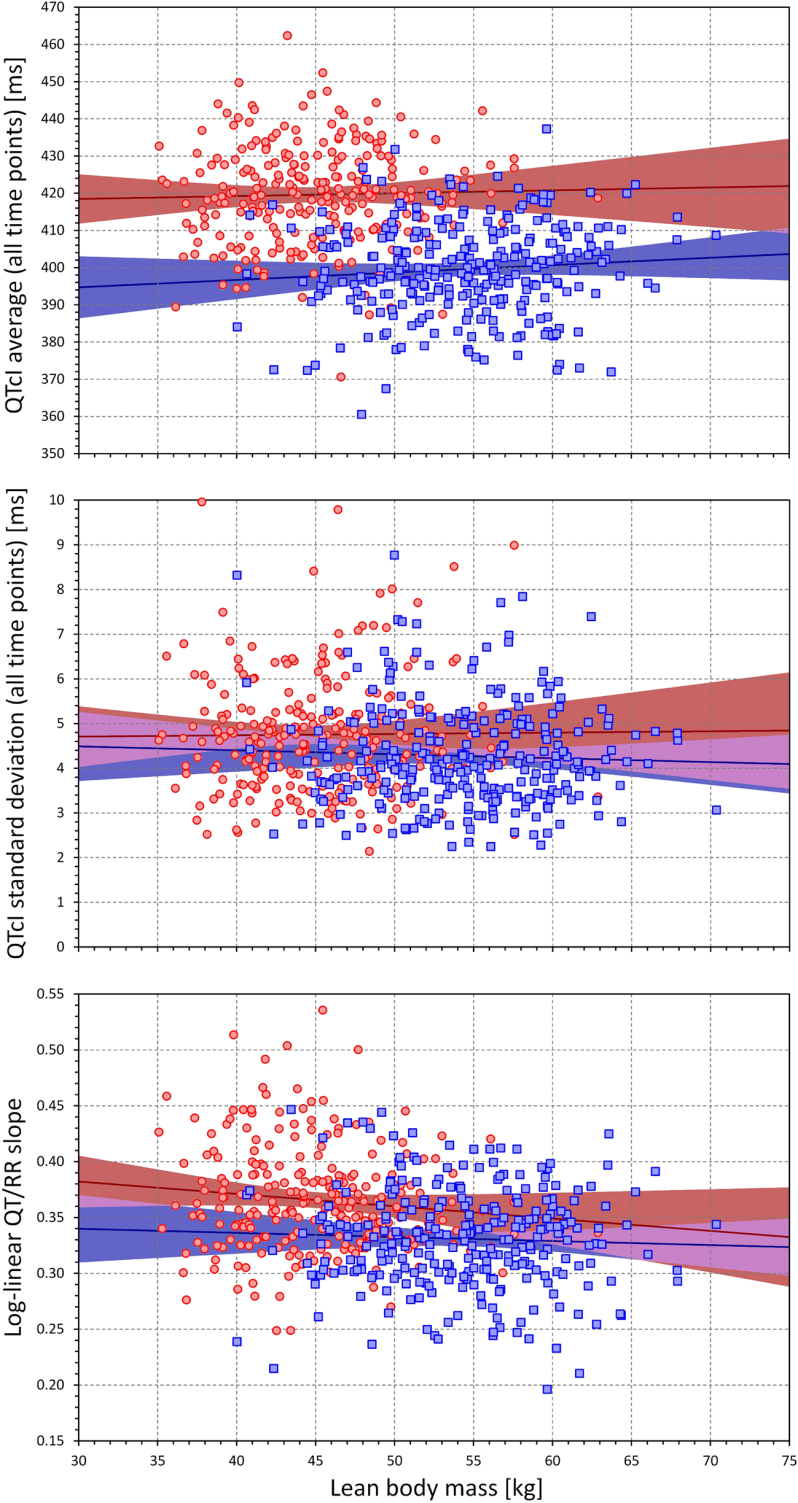
Scatter diagrams between the lean body mass and mean QTcI values of all ECG measurements in all study time-points (top panel), standard deviation of QTcI values of all ECG measurements in all study time-points (middle panel), and subject-specific log-linear QT/RR slope (bottom panel). The meaning of symbols and Figure layout is the same as in Fig. 3. *kg* kilograms, *ms* milliseconds.

Figures 3, 4 and 5 show the relationship between the individual QT/RR characteristics derived from ECG measurements in all study time-points and age, BMI, and LBM. While different trends can be detected in these scatter diagrams, the only significant relationships were the QTcI prolongation with advancing age (*p* = 0.01 in both sexes), decrease with QT/RR slope with age in males (*p* = 0.009, NS in females), and decrease in QT/RR slope with increased BMI (*p* = 0.011 and *p* = 0.016 in females and males, respectively). Despite expectations, we have not found any significant influence of age, BMI, or LBM on the intra-subject SD of QTcI.

### Short-term QTc variability

**Figure 6:**
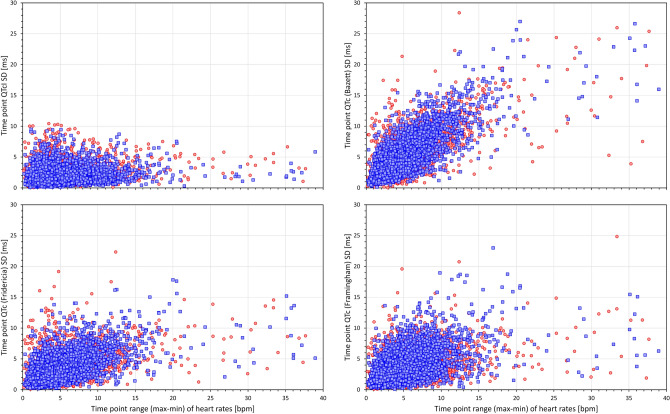
Scatter diagrams between heart rate ranges (maximum–minmum) and standard deviations of QTc values of individual study time-points. The left top panel shows the relationship for QTcI intervals, the top right, bottom left, and bottom right panels show the relationship for Bazett, Fridericia, and Framingham corrected QTc intervals, respectively. In all panels, all time-points of all study subjects are pooled together, red and blue marks show the data of female and male subjects, respectively. Outliers beyond the ranges of the axes were also present in the data. Note that pooling all time-points of all subjects together is not suitable for statistical analysis but serves visual interpretation—the stronger the relationship between the variability of the underlying heart rate and the variability of the QTc intervals within the same time-point, the greater the failure of the correction formula in eliminating the effects of heart rate on QTc values. Compare the panels with panels of Fig. 7. *bpm* beats per minute, *max* maximum, *min* minimum, *SD* standard deviation.

**Figure 7:**
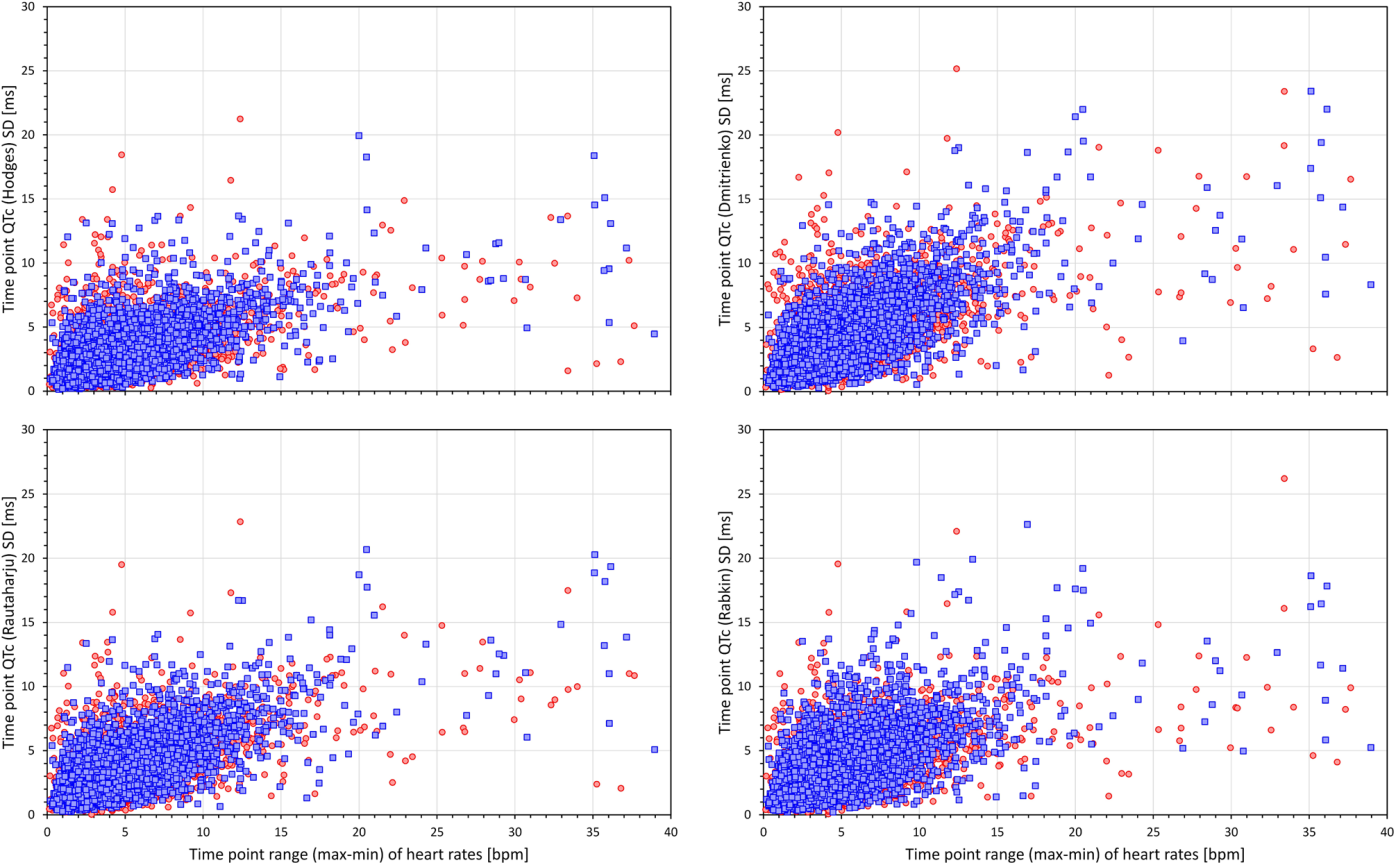
Scatter diagrams between heart rate ranges (max–min) and standard deviations of QTc values of individual study time-points. The left top, top right, bottom left, and bottom right panels show the relationship for Hodges, Dmitrienko, Rautaharju, and Rabkin corrected QTc intervals, respectively. In all panels, all time-points of all study subjects are pooled together, red and blue marks show the data of female and male subjects, respectively. Outliers beyond the ranges of the axes were also present in the data. The abbreviations and meaning of panels are the same as in Fig. 6. The note in the caption of Fig. 6 also applies—compare the panels with panels of Fig. 6.

Figures 6 and 7 show scatter diagrams that relate 5-min SDs of QTc values measured within the same study time-point to the range (max–min) of heart rates among the same 10-s ECG samples in which the QTc values were measured. This relationship is shown for the QTcI values (top left panel of Fig. 6) and for 7 of the investigated correction formulas. The figures show that while the short-term variability of the QTcI values is little influenced by the heart rate ranges, the short-term variability of heart rates of the measured ECG samples influenced the short-term variability of all the QTc formulas with the strongest relationship visible for the Bazett formula.

**Figure 8:**
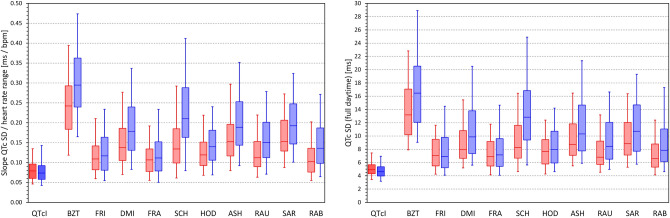
The left panel shows the distribution of the intra-subject linear slopes between heart rate ranges (max–min) and standard deviations of QTc intervals calculated over the different study time-points. The right panel shows the distribution of the intra-subject standard deviations of the QTc intervals calculated over all the ECG readings within the drug-free baseline day. For each correction formula (see the abbreviations at the horizontal axes) red and blue box and whisker entries are shown corresponding to the distribution of the data in female and male subjects, respectively. Each of the box and whisker entries shows the population median (horizontal line within the box), inter-quartile range (the top and bottom of the box) and the range between the 5th and 95th percentile (the bottom and top whiskers). *bmp* beats per minute, *ms* milliseconds, *SD* standard deviation.

The data summarised in the images of Figs. 6 and 7 are not suitable for statistical analysis since for each subject, the scatter diagrams contain data of multiple study time-points. Data suitable for statistical analysis are shown in the left panel of Fig. 8. This panel of Fig. 8 shows, for each heart rate correction, the spread of linear regression slopes between the 5-min SDs of QTc values and the corresponding 5-min heart rate ranges. In each subject, the regression was calculated over all study time-points. While for QTcI, the displayed slopes were not significantly different between females and males, the slopes were significantly lower in females compared to males for all correction formulas (*p* = 0.0347 for Fridericia formula, *p* = 0.0079 for Framingham formula, *p* < 0.00001 for all other formulas). Paired comparison between the formulas showed that (a) the QTcI slopes were smaller than for any other correction formula (*p* < 0.00001 for all comparisons), (b) the slopes of Bazett formula were larger than for any other formula (*p* < 0.00001 for all comparisons), (c) the slopes of Fridericia and Framingham formula were only little different of each other, and (d) the slopes of all other formulas were larger compared to the slopes and Fridericia and Framingham formulas (*p* < 0.00001 for all comparisons).

### Day-time QTc variability

For comparisons of results discussed in the previous section, the right panel of Fig. 8 shows the distribution of SDs of QTc values over all the ECG readings available during the baseline day. It is well visible that the patterns of the two panels of Fig. 8 closely replicate each other. Indeed, the data led to the very same statistical comparisons: For all formulas, the SDs of QTcI were significantly smaller (*p* < 0.00001 for all), SDs of Bazett QTc were larger than any other formula (*p* < 0.00001 for all), SDs of Fridericia and Framingham QTc were not significantly different, and SDs of Fridericia and Framingham were significantly smaller than those of any other formula (*p* < 0.00001 for all comparisons).

**Figure 9:**
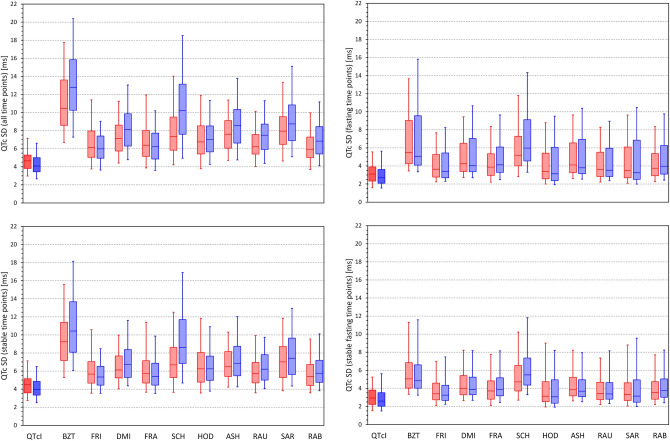
Distribution of the intra-subject standard deviations of QTc values calculated over all study time-points (top left panel), time-points with stable heart rate (bottom left panel), morning fasting time-points (top right panel), and morning fasting time-points with stable heart rate (bottom right panel). The layout of the panels and the meaning of the box and whisker graphs is the same as in Fig. 8. *ms* milliseconds, *SD* standard deviation.

Figure 9 shows the distributions of SDs of QTc values obtained from all study time-points, stable time-points, fasting time-points, and fasting stable time-points. The QTc variability values decreased in this order of time-point selection. For instance, for SDs of QTcI values, the corresponding population means of the SDs (females and males combined) were 4.50, 4.38, 3.17, and 3.06 ms, respectively (*p* < 0.002 for statistical comparisons at all individual steps). For Bazett correction, the corresponding values were 12.14, 10.37, 6.90, and 5.85 ms (*p* < 0.00001 at all steps) while for Fridericia correction, the corresponding values were 6.42, 5.87, 4.20, and 3.86 ms (*p* < 0.00001 at all steps). Systematically, the SDs of the Bazett QTc values were significantly larger than those of any other formula (*p* < 0.00001 for all comparisons) and those of Fridericia correction were smaller than those of any other formula (*p* values ranged between 0.033 and < 0.00001 for all comparisons).

### Difference between general and individual QTc corrections

**Figure 10:**
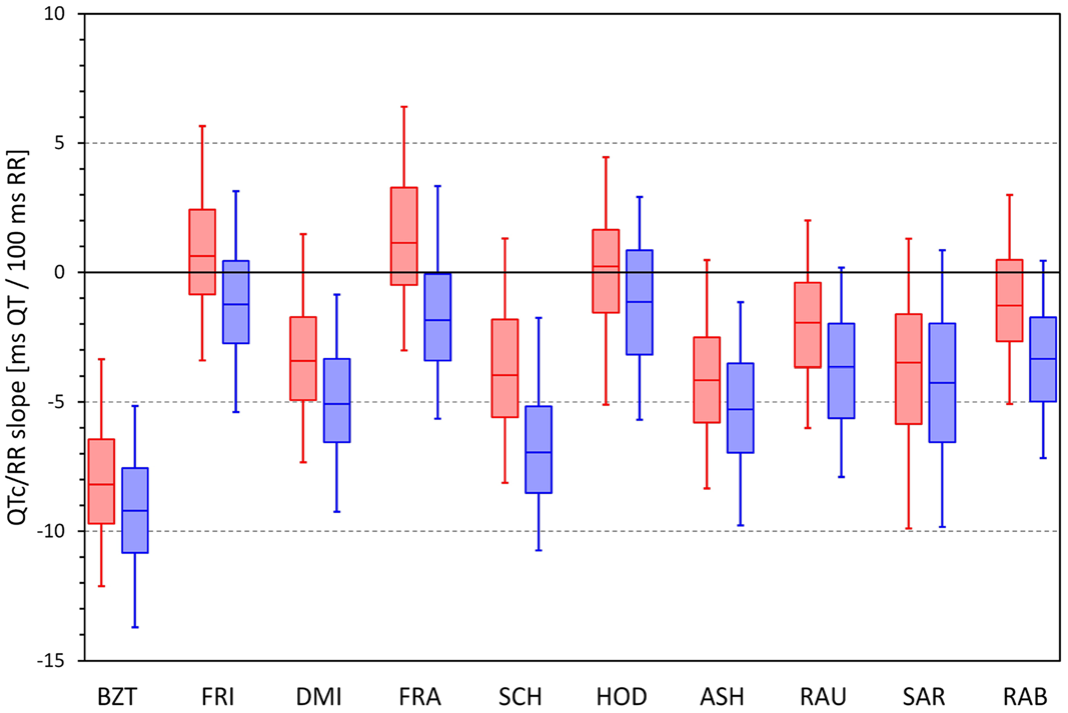
Distribution of intra-subject linear slopes between QTc values and corresponding 10-s averages of RR intervals calculated over all ECG reading during the baseline drug-free day. The layout of the panel and the meaning of the box and whisker graphs is the same as in Fig. 8. *ms* milliseconds.

**Figure 11:**
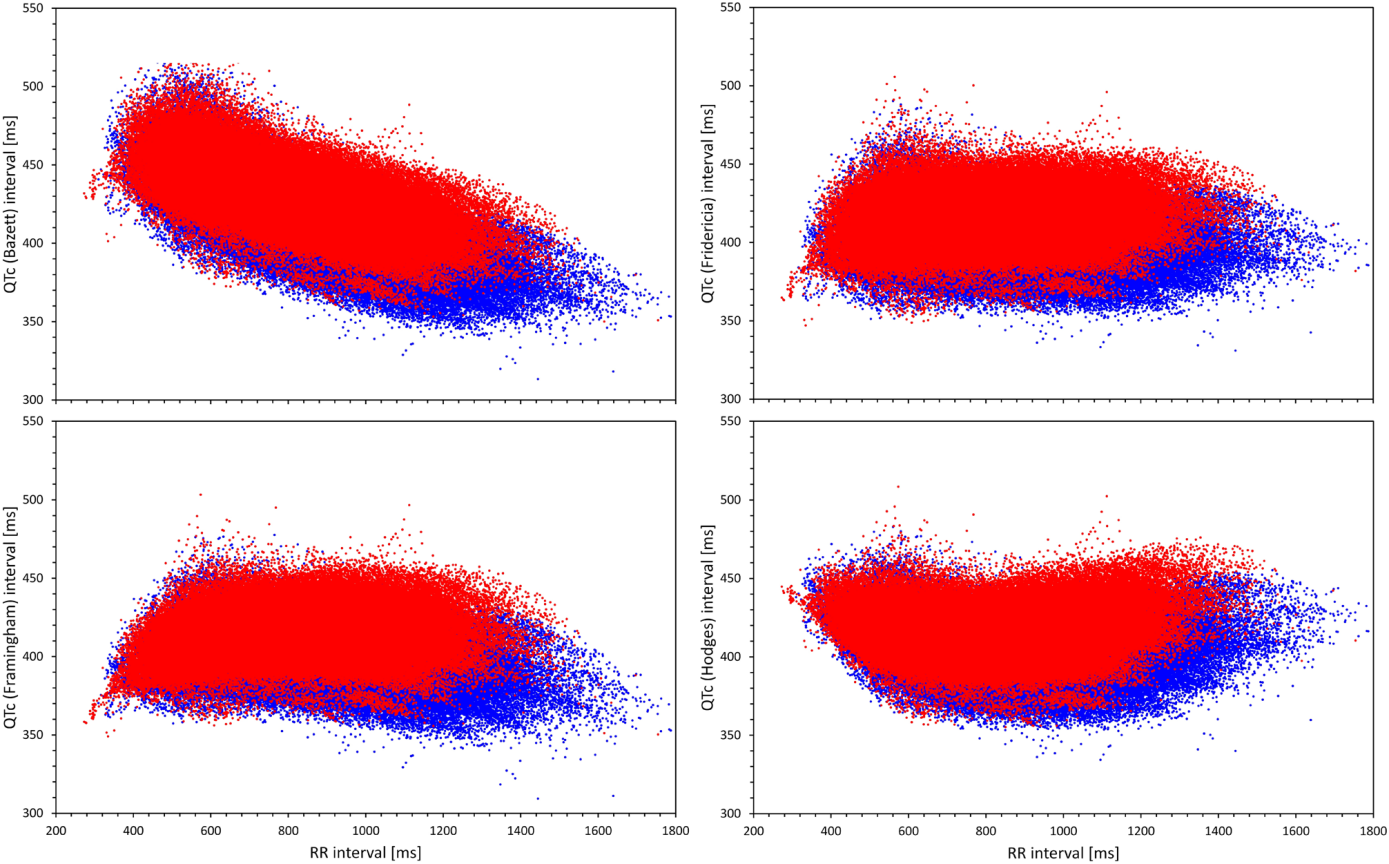
For Bazett (top left panel), Fridericia (top right panel), Framingham (bottom left panel) and Hodges (bottom right panel) correction, the Figure shows scatter diagrams of QTc values versus underlying heart rate (10-s measurement) in all study data pooled together. The data in females (in red) are shown superimposed on top of the data in males (in blue).

Figure 10 shows the distribution of slopes of intra-subject linear regressions between QTc values and corresponding 10-s averages of RR intervals. Very clearly, Bazett correction led to QTc values that were least independent of the underlying RR intervals (i.e. led to the poorest elimination of the QTc dependency on the underlying heart rate) while Fridericia and Hodges corrections led to slopes that were, on average, closest to zero. (Understandably, the figure does not show the results for QTcI since the individual corrections are designed to produce 0 slopes.) The fact that, compared to other correction, Bazett formula leads to QTc values that are not independent of the underlying heart rate is also visible when pooling individual ECG data of all subjects together (Fig. 11).

**Figure 12:**
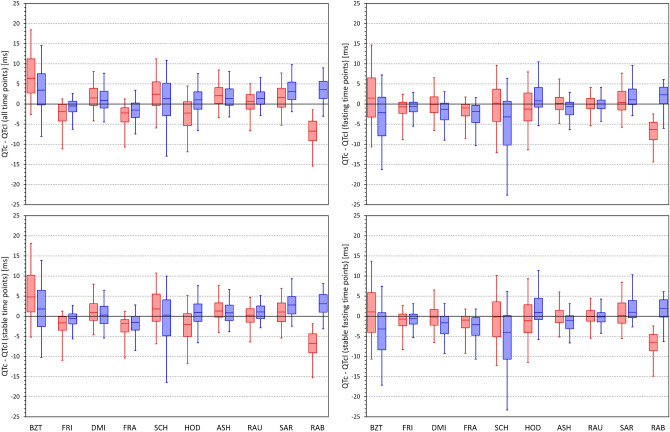
Distribution of the mean intra-subject differences between QTc intervals by different correction formulas and the corresponding QTcI values. The distribution of the differences is shown for calculations over all study time-points (top left panel), time-points with stable heart rate (bottom left panel), morning fasting time-points (top right panel), and morning fasting time-points with stable heart rate (bottom right panel). The layout of the panels and the meaning of the box and whisker graphs is the same as in Fig. 8. *ms* milliseconds.

Figure 12 shows the distribution of the differences between QTcI values and the QTc values by different correction formulas for all study time-points, stable time-points, fasting time-points, and fasting stable time-points. For each Formula, there is an appreciable spread of the differences but the largest is seen for Bazett and Schlamowitz corrections. The figure also shows the attempt of eliminating QTc differences between females and males and eliminating the age difference by Rabkin et al. leads to systematic bias of the formula, especially in females.

**Figure 13:**
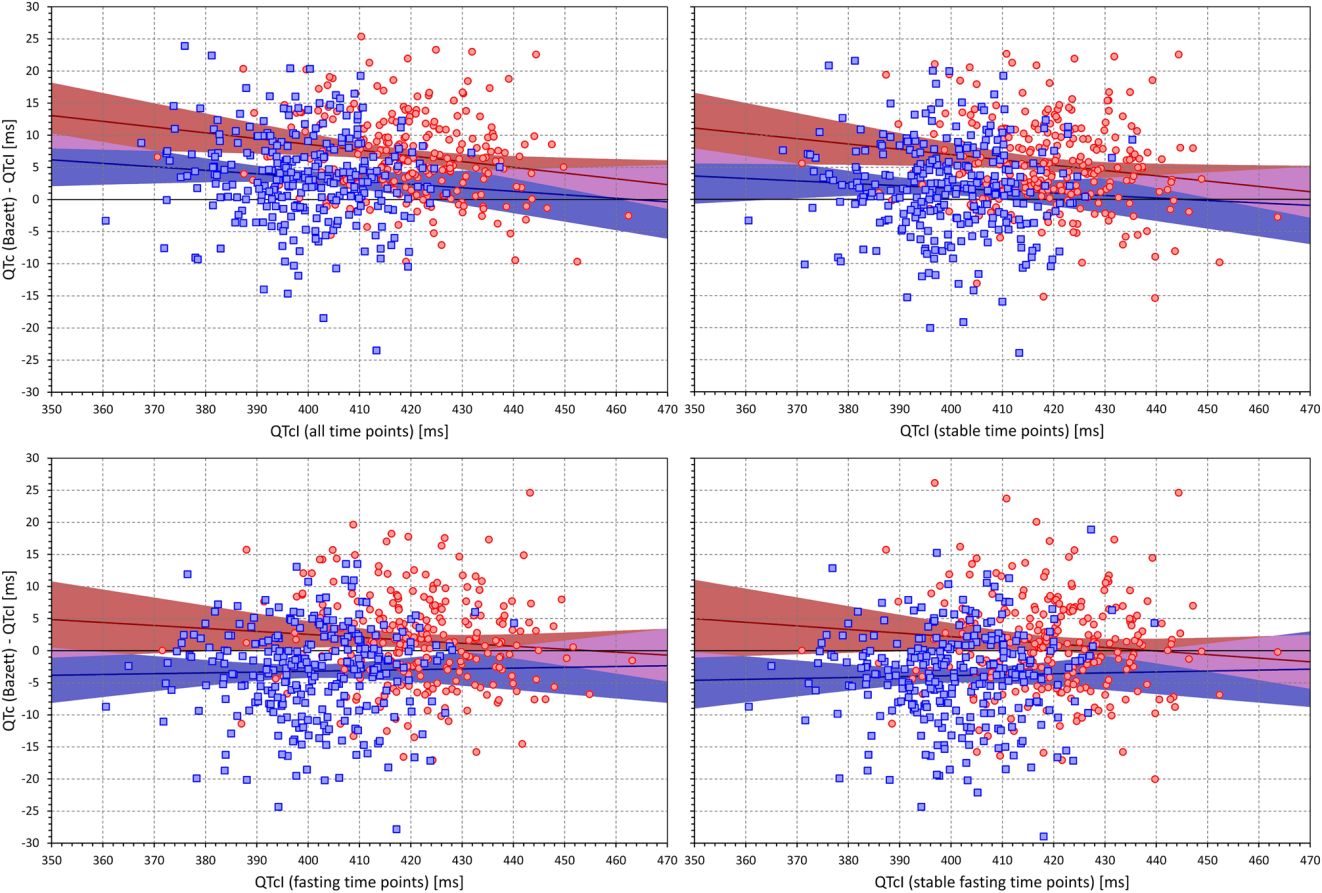
Scatter diagrams of the mean intra-subject differences between Bazett corrected QTc intervals and corresponding QTcI intervals plotted against the intra-subject means of QTcI intervals. The diagrams are shown for data of all study time-points (top left panel), time-points with stable heart rate (top right panel), morning fasting time-points (bottom left panel), and morning fasting time-points with stable heart rate (bottom right panel). In each panel, the red circles and blue squares correspond to female and male subjects, respectively. The solid red and solid blue lines show the linear regressions between the intra-subject QTcI means and the mean intra-subject QTc – QTcI differences in females and males, respectively. The red shaded and blue shaded areas are the 95% confidence intervals of the regression lines; the violet areas are the overlaps between the confidence intervals of the sex-specific regressions. *ms* milliseconds.

**Figure 14:**
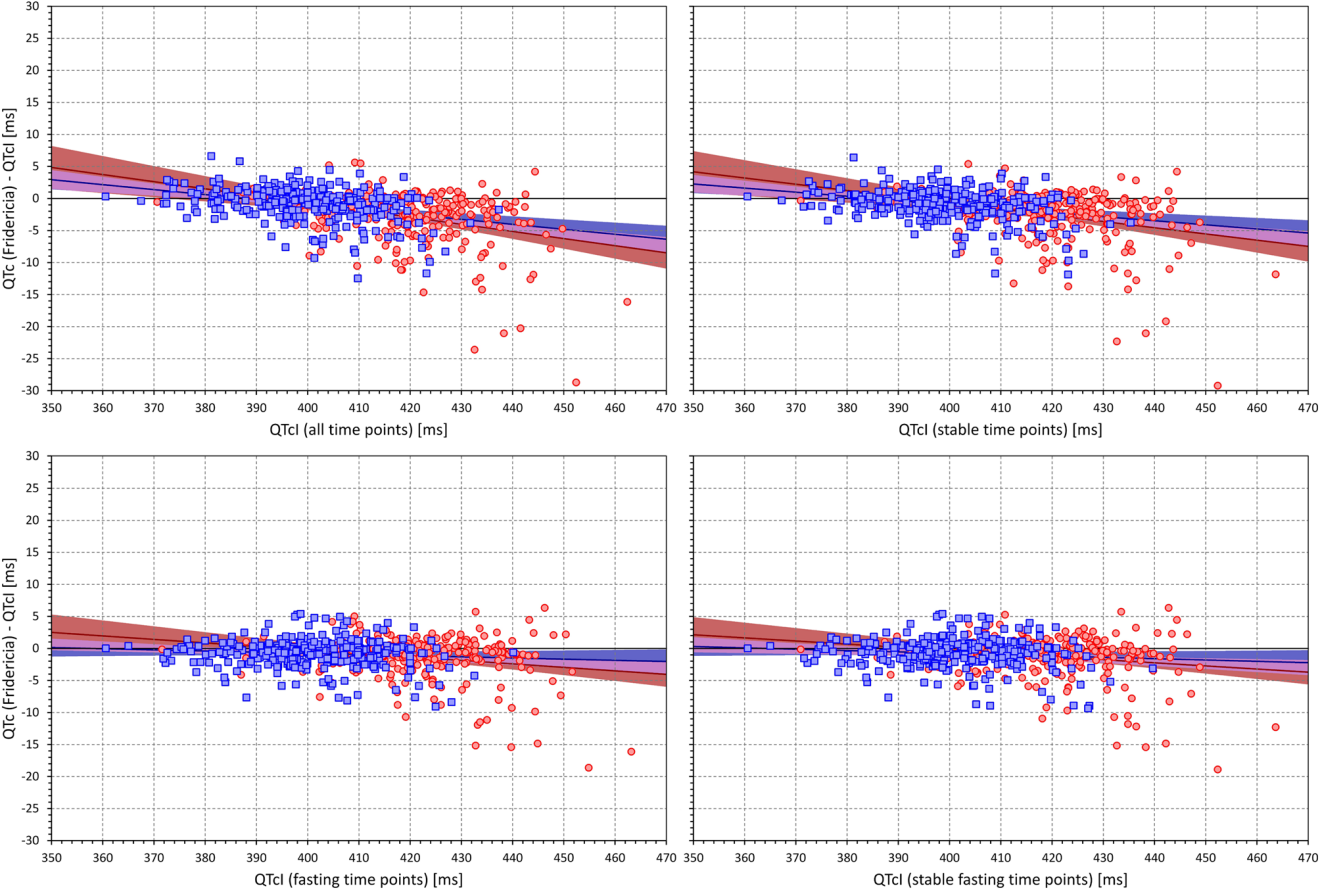
The figure meaning and layout correspond to those of Fig. 12 but instead Bazett QTc data, Fridericia QTc data were used.

**Figure 15:**
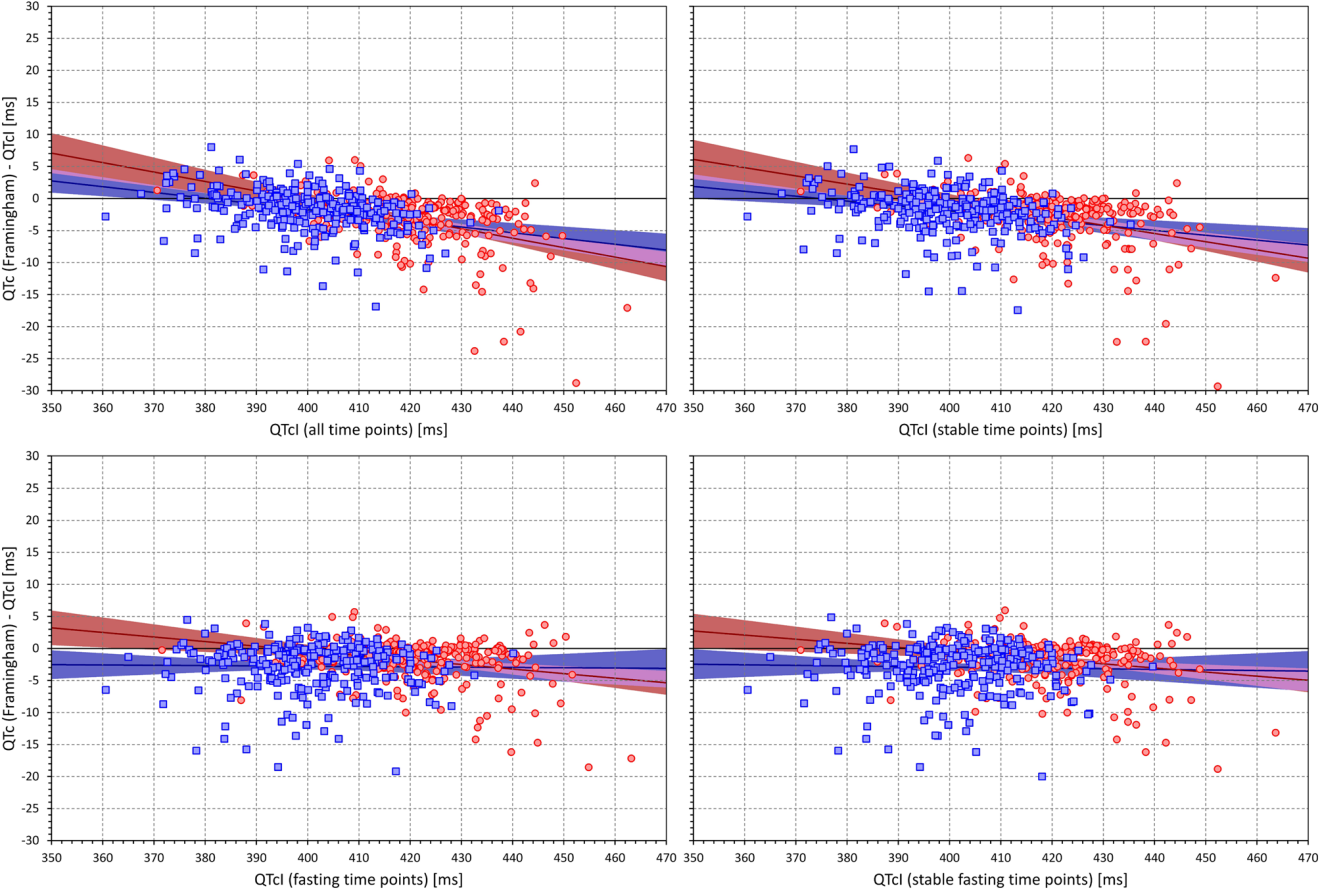
The figure meaning and layout correspond to those of Fig. 12 but instead Bazett QTc data, Framingham QTc data were used.

**Figure 16:**
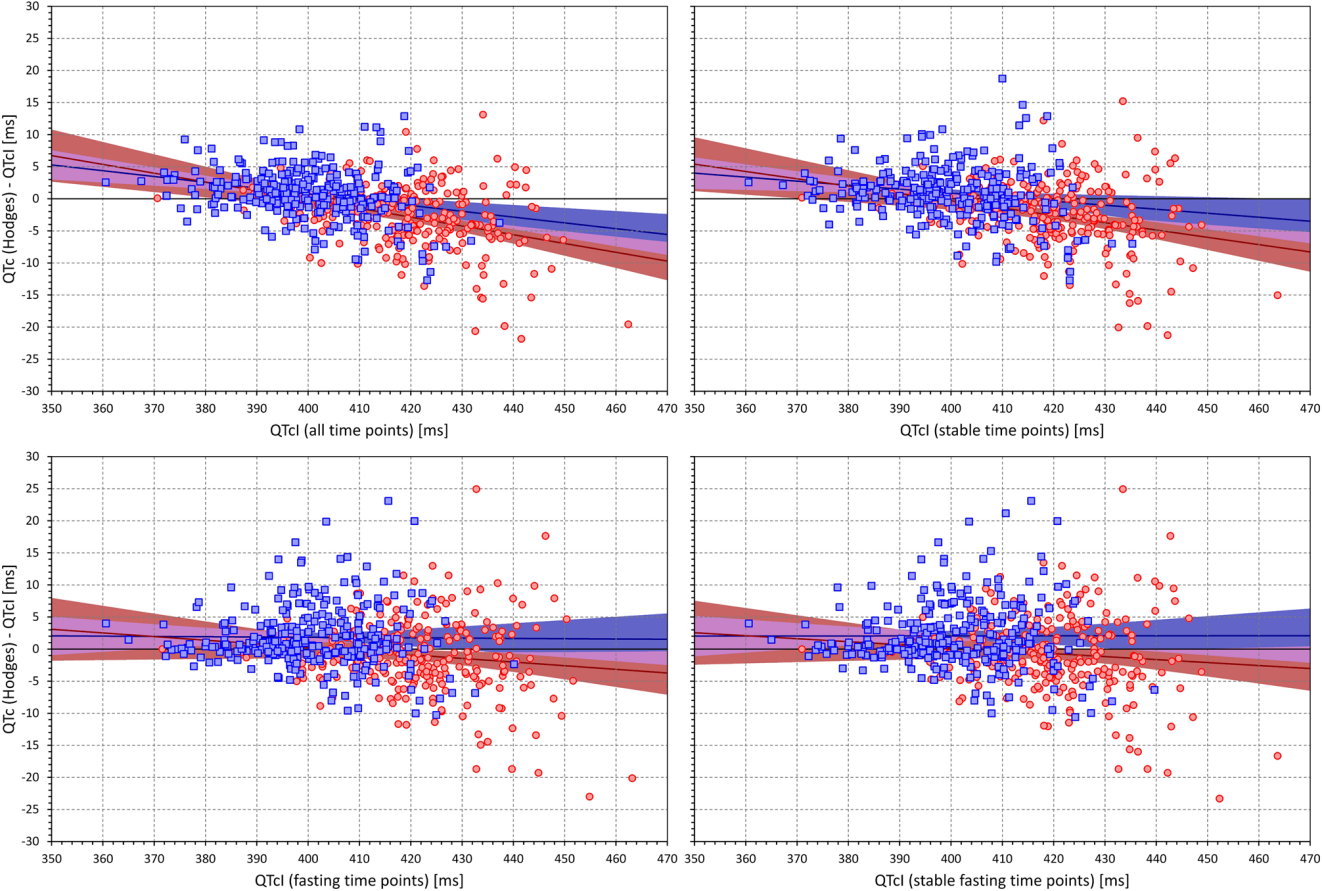
The figure meaning and layout correspond to those of Fig. 12 but instead Bazett QTc data, Hodges QTc data were used.

Importantly, Fig. 13 shows that when considering data of all study time-points, there was a systematic trend between the QTcI values and the QTc (Bazett)—QTcI differences. This trend was persistent also when considering only stable time-points but it was reduced when analysing only fasting time-points and further reduced when considering the stable fasting time-points. Figures 14, 15 and 16 show that this observation was also present for other formulas (the Figures show the details for Fridericia, Framingham, and Hodges corrections).

## Discussion

### Principal observations

Four principal observations may be derived from the study; all with potentially important implications for clinical electrocardiography.

Whilst this is not the first study to document substantial problems with Bazett correction^34,35,37,39,41,54–56^, we show that this correction not only fails to remove the relationship between QTc intervals and the underlying heart rate, it also leads to QTc data that are much more variable compared to all the other corrections that we investigated. We recognise that Bazett correction became somewhat entrenched in clinical reporting, possibly because historically it was very easy to implement and calculate. With the widespread advances of technical possibilities, this superficial advantage has been lost. We are therefore of the strong opinion that all the previous criticism of Bazett formula should finally be heard. The clinical practice needs to be changed and Bazett correction should stop being used. This would not only improve the assessment of QTc interval in clinical cases but would also correspond to the regulatory evaluation of QTc interval in clinical drug studies in which Bazett correction has now been abandoned^20^.

Obviously, a generic correction formula is needed in clinical practice since, as already stated, individual-specific baseline QT/RR profile cannot possibly be obtained for clinical purposes (investigations over full drug-free day or days are needed for this purpose in clinical pharmacology studies^25,26,57^). The overall comparison of the 10 formulas that we investigated give a clear preference to Fridericia or Framingham corrections that not only lead to lesser variable results compared to the other formulas but, in our data, appeared not very far from the QTcI values. The argument that the presently available clinical experience based on Bazett correction cannot be easily applied to these corrections needs to be refuted. For heart rates close to 60 bpm, the results of all the corrections are the same and heart rate departures from 60 bpm make Bazett QTc values polluted by larger errors compared to the other corrections. Contrary to Framingham formula, Fridericia formula suffers from non-linearity^58^ (also present with Bazett formula) which makes it much less accurate with other ECG intervals (e.g. the JT interval). Nevertheless, when applied to the physiologically plausible QT durations, the effects of this problem are not large. Recently, we have also found preference for Fridericia and Framingham formulas when assessing QTc duration in school-aged children^59^.

While “normalisation” attempts by the Rabkin formula may lead to homogeneity of QTc values across a population of both sexes, the bias of the formula seen in Fig. 11 makes its usefulness very limited for any practical purposes. The artificial reduction of QTc in females as well as in subjects of advanced age means that in clinical use, the formula would compromise detection of susceptibility to drug-induced QTc prolongation (known to be increased in females^60^) and of reduced repolarisation reserve (known to be more frequent with advanced age^61,62^). Thus, while the reproducibility of the QTc by Rabkin formula was comparable to that of Fridericia and Framingham formulas (see Fig. 9), the comparison with the QTcI values showed that for practical use, this formula would be reproducibly “off target”.

The situations of heart rate instability effects demonstrated in Figs. 1, 6 and 7 need to be considered carefully. It has already been discussed that positioning subjects in motionless position might reduce heart rate changes due to physical demands but cannot eliminate heart rate instability due to other (e.g., psychological) factors^46^. For clinical practice, we would like to suggest that repeated ECG recordings are obtained within an interval of some (e.g., 3 to 5) minutes and that QTc interval is only derived from these recordings if they all show similar heart rate (e.g., within a 5 bpm range). To be properly understood: we are not advocating that multiple QTc interval measurements are averaged to reduce the QTc variability. Such an approach would not eliminate the potentially large errors due to the effects of QT/RR hysteresis. We suggest that multiple ECG recordings should be used to assure that the ECG in which the QT interval is measured (e.g. the last of the repeated recordings) is preceded by stable heart rate. As shown in the example of Fig. 1, variable heart rate preceding the QT measurement can invalidate QTc assessment well above the inaccuracy of generic correction formulas. The fact that the duration of the profile of QT/RR hysteresis, i.e. the time needed for QT interval to adapt to changing heart rate, is longer in cardiac patients compared to healthy subjects^63^ makes the considerations of QT/RR hysteresis even more important for clinical practice. Seeking heart rate stability over the interval of some minutes is bound to supress gross errors of QTc assessment.

Finally, although we included several correction formulas that have been proposed more recently and utilise fairly complex mathematical forms (e.g. the definition of the spline formula used in the correction by Rabkin et al. occupies 27 lines of complex mathematical text^42^), we have not found these advantageous in comparison to the older and also much simpler Fridericia and Framingham corrections. We therefore believe that efforts to design yet another correction formula for general use are fruitless and will not lead to anything substantially more accurate and/or more stable than Fridericia and/or Framingham corrections.

### Physiologic comments

It is not surprising that none of the investigated general correction produces data very close to subject-specific corrections. This is because, in principle, no correction can exist that would faithfully reproduce the QT/RR relationship in all subjects and patients^51,64^. This is well documented in the images in Fig. 2. If we consider RR interval changes between 600 and 900 ms, it can be seen in this Figure that some subjects change their physiologic QT interval durations by as little as 15 ms while in other subjects, the corresponding QT interval change exceeds 30 ms. Therefore, there cannot be a mathematical form (regardless of how complicated) that would correctly model all these cases. The best of what can be achieved is to find a mathematical form that is reasonably close to the physiologic “middle” of different QT/RR patterns and that will, correspondingly, be overestimating and underestimating the true QTc values with equal or similar frequencies. Since the individual QT/RR patterns differ not only in their slopes but also in their curvatures^30^, even substantial complexity of mathematical forms would not be too helpful. For the reasons of substantial individuality of QT/RR adaptation, proposals of different formulas for females and males are also of little value (despite the statistical sex differences in QTc duration and QT/RR slope^52^). There is a very substantial overlap in the subject-specific QT/RR patterns and slopes between both sexes^46^.

Because of the substantial spread of intra-individual QT/RR patterns, it seems problematic to diagnose QTc abnormality based on ECG recordings with heart rate very remote from the 60 bpm “centre”. This is particularly true for the Bazett formula while Fridericia and Framingham formulas are somewhat more robust when used to assess QTc duration at heart rates between, say, 55 and 75 bpm^65^. Within such heart rate ranges, the “standard” abnormal prolongation cut-offs (e.g. 470 and 450 ms for females and males, respectively) might be used also with these formulas since the population spread of their QTc values is not very far from that at 60 bpm^65^.

It might also be speculated that in addition to heart rate and QT/RR hysteresis effects, QT interval might also be corrected for other factors such as autonomic status and/or plasma electrolyte and glucose levels^43,66,67^. In this sense, our “gold standard” individual correction might be further improved. Nevertheless, as previously shown and as also demonstrated in our present results, the intra-subject variability of QTcI values is fairly low^64,68^ and considerations of rate-independent QT covariates might only lead to minimal advances.

Contrary to individuality of QT/RR adaptation patterns (i.e. how much does the QT interval change in response to different heart rate) the QT/RR hysteresis profiles (i.e. how quickly the QT interval reacts to heart rate changes) are similar in different healthy subjects. Hence, while in different healthy subjects, even Fridericia and Framingham formulas may lead to noticeable differences between QTc and QTcI (see, Figs. 13 and 14) the use of universal hysteresis correction leads only to minimal departures from individually optimised QT/RR hysteresis models of healthy subjects^46^. As already mentioned, QT/RR hysteresis is slower in cardiac patients^63^, but it is not known whether the previously proposed universal model of QT/RR hysteresis correction would lead to substantial discrepancies in the QTc assessment in such patients. Nevertheless, for clinical practice, these considerations are rather academic since the application of hysteresis correction requires the data of a prolonged history of QT measurement, well exceeding the standard 10-s ECG duration. Therefore, elimination of the need to correct for QT/RR hysteresis needs to be the method of choice. This leads to the assurance of heart rate stability as we have already proposed. It remains to be investigated whether the rate stability limit of 5 bpm that we have used might be proposed for clinical electrocardiography or whether different criteria should be applied, especially when recordings patients with cardiac abnormalities.

Comment should also be made on the fact that we observed both short-term and long-term variations not only of QTc values by the different correction formulas but also, albeit to a much lesser degree, of the individually corrected QTcI values. While direct heart rate influence on the QTcI duration is removed, ventricular repolarisation interval is also under the influence of autonomic nervous system^67^ as well as of other regulatory mechanisms, e.g. those responsible for post-prandial effects^43^. This means that even at the same underlying heart rate, differences in the QT interval duration still exist (note the “width” of the QT/RR patterns shown in Fig. 2). The dual influence of autonomic reflexes on both heart rate and QT interval duration is not necessarily in synchrony and thus, short-term QTcI variability might be more pronounced during episodes of increased autonomic and thus also heart rate fluctuations. Indeed, we have recently reported that increased heart rate and increased short-term RR interval variability are significant contributors to the beat-to-beat QT interval variations^69^. The autonomic influence of QTcI intervals also contributes to the observed increased mean QTcI with advancing age. Similarly, the decline of the QT/RR slope with advancing age and with increased BMI and LBM might be attributed to the decline of autonomic responsiveness which is known not only with an increased age but also with changes in body habitus.

### Limitations

The study used data of healthy volunteers rather than of patients in whom QTc monitoring is needed for clinical reasons. Nevertheless, while the inter-subject spread of QT/RR patterns might be wider among patients with different diagnoses compared to healthy subjects, it can hardly be narrower. Hence, a correction formula that performs poorly in healthy subjects is very unlikely to operate more reasonably in a patient population.

Some of the previous reports tried to select an optimum formula not only for accurate rate correction of the QT interval (that is, for obtaining QTc values independent of heart rate) but also for other purposes, such as the prediction of poor survival and/or arrhythmic risk^22^. We have not addressed such approaches since they are, in principle, methodologically flawed. Increased heart rate is a known risk factor in its own right^70,71^ and trying to incorporate its risk predictive influence into a QT correction formula is both physiologically and clinically problematic. If risk prediction is needed involving both QTc interval duration and heart rate, multivariable stratification models are required.

The 10 correction formulas that we have investigated have been selected from a very broad spectrum of other proposals. We believe that the formulas that we selected cover the spectrum of previously published correction methods reasonably and that it would be fruitless to include also other possibilities. When we experimented with several other formulas not included in this study, the results (not shown here) were fully consistent with the observations that we described.

In the setup of “gold standard” QTcI values, we used the subject-specific QT/RR curvature modelling combined with the exponential decay QT/RR hysteresis model^30,31^. The variability of the QTcI might have been further reduced not only by employing more complex hysteresis corrections^72–74^ but also by differentiating the speed of QT adaptation to heart rate acceleration and deceleration. As far as we are aware, such a distinction of different QT/RR hysteresis components has never been attempted before. Likewise, it might be possible to correct QT interval not only for underlying heart rate but also for the spectral components of heart rate variability that approximate the sympatho-vagal balance of heart rate modulations. Again, we are not aware of any technology available for such a purpose although the different levels of sympatho-vagal modulations are likely to influence QT interval differently even at the same level of the underlying heart rate. In any case, however, only miniscule increases of QTcI stability might be achieved by all these approaches since the variability that we reported was already very low.

### Conclusion

The principal conclusions of the study might be summarised as follows:

Our observations fully support the previous criticism of Bazett correction. In all investigated aspects, this correction performed more poorly compared to all other possibilities. A repeated strong call to the clinical community is needed so that this correction is finally eliminated from everyday use.

Among the broad spectrum of correction formulas that we investigated, most stable and (on average) reasonably accurate QTc corrections were obtained with Fridericia and Framingham corrections. While for research and investigative use, these corrections are less suitable than the subject-specific optimisation approaches, clinical practice would substantially benefit from using either of these corrections instead of the Bazett formula.

To eliminate potentially large QTc inaccuracies due to preceding variable heart rate, repeated ECG recordings within a short interval and some minutes should be obtained and the heart rate stability objectively verified.

Further attempts of finding a universally applicable correction formula that would accurately correct QT intervals in different conditions appear unproductive and clearly impossible to succeed because of the large physiologic spread of individual QT/RR profiles.

## Data Availability

The raw data supporting the conclusions of this article will be made available by the authors, without undue reservation but pending the approval by the sponsors of the source clinical studies.
